# α-NH_4_Fe(HAsO_4_)_2_

**DOI:** 10.1107/S1600536814008691

**Published:** 2014-05-03

**Authors:** Najoua Ouerfelli, Amira Souilem, Mohamed Faouzi Zid, Ahmed Driss

**Affiliations:** a Laboratoire de Matériaux et Cristallochimie, Faculté des Sciences de Tunis, Université de Tunis ElManar, 2092 Manar II Tunis, Tunisia

## Abstract

The title compound α-ammonium iron(III) bis­[hydro­gen arsenate(V)], α-NH_4_Fe(HAsO_4_)_2_ (or poly[ammonium bis­(μ-hydro­gen arsenato)ferrate(III)], {NH_4_[Fe(HAsO_4_)_2_]}_*n*_), syn­the­sized hydro­thermally, is isostructural with NH_4_Fe(HPO_4_)_2_. Condensation of the hydro­gen arsenate groups with FeO_6_ coordination octa­hedra *via* common corners results in an overall three-dimensional framework containing inter­connected channels parallel to the *a-*, *b-* and *c*-axis directions. The NH_4_^+^ cations are located in three inter­secting tunnels, which is promising as an ion exchange. Hydrogen bonding of the types O—H⋯O and N—H⋯O consolidates the packing of the structure. The distortion of the coordination polyhedra is analyzed by means of the effective coordination number and distortion indices. Structural relationships with other compounds of general formula *M* ^I^ *M* ^III^[H*X*O_4_)]_2_ (*X* = P, As) are discussed.

## Related literature   

For related compounds and structures, see: Yakubovich (1993 ▶); Lesage *et al.* (2007 ▶); Lii & Wu (1994 ▶); Haushalter *et al.* (1995 ▶); Bircsak & Harrison (1998 ▶); Stalder & Wilkinson (1998 ▶); Yan *et al.* (2000 ▶); Schwendtner & Kolitsch (2004*b* ▶). For similar materials, see: Huang *et al.* (2004 ▶); Ewald *et al.* (2004 ▶); Filaretov *et al.* (2002 ▶); Guesdon *et al.* (2009 ▶); Gurbanova *et al.* (2001 ▶); Menezes *et al.* (2008 ▶, 2009*a* ▶,*b* ▶); Mao *et al.* (2002 ▶); Schwendtner & Kolitsch (2004*a* ▶); Vencato *et al.* (1989 ▶); Villars *et al.* (2007 ▶). For ECoN and ID distortion parameters, see: Baur (1974 ▶); Wildner (1992 ▶); Nespolo (2001 ▶); Nespolo *et al.* (2001 ▶). For bond-valence sums, see: Brown & Altermatt (1985 ▶).

## Experimental   

### 

#### Crystal data  

(NH_4_)Fe(HAsO_4_)_2_
*M* *_r_* = 353.75
Triclinic, 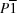

*a* = 7.3473 (7) Å
*b* = 9.1917 (8) Å
*c* = 9.7504 (9) Å
α = 64.545 (5)°
β = 70.710 (7)°
γ = 69.638 (6)°
*V* = 544.55 (9) Å^3^
*Z* = 3
Mo *K*α radiation
μ = 11.14 mm^−1^
*T* = 298 K
0.21 × 0.13 × 0.09 mm

#### Data collection  

Enraf–Nonius CAD-4 diffractometer
Absorption correction: ψ scan (North *et al.*, 1968 ▶) *T*_min_ = 0.187, *T*_max_ = 0.388
2737 measured reflections
2373 independent reflections
2121 reflections with *I* > 2σ(*I*)
*R*_int_ = 0.021
2 standard reflections every 120 min intensity decay: 1.1%

#### Refinement  

*R*[*F* ^2^ > 2σ(*F* ^2^)] = 0.027
*wR*(*F* ^2^) = 0.075
*S* = 1.05
2373 reflections
197 parameters
8 restraints
H atoms treated by a mixture of independent and constrained refinement
Δρ_max_ = 1.31 e Å^−3^
Δρ_min_ = −1.21 e Å^−3^

### 

Data collection: *CAD-4 EXPRESS* (Duisenberg, 1992 ▶; Macíček & Yordanov, 1992 ▶); cell refinement: *CAD-4 EXPRESS*; data reduction: *XCAD4* (Harms & Wocadlo, 1995 ▶); program(s) used to solve structure: *SHELXS97* (Sheldrick, 2008 ▶); program(s) used to refine structure: *SHELXL97* (Sheldrick, 2008 ▶); molecular graphics: *DIAMOND* (Brandenburg & Putz, 2001 ▶); software used to prepare material for publication: *WinGX* (Farrugia, 2012 ▶) and *publCIF* (Westrip, 2010 ▶).

## Supplementary Material

Crystal structure: contains datablock(s) I. DOI: 10.1107/S1600536814008691/br2238sup1.cif

Structure factors: contains datablock(s) I. DOI: 10.1107/S1600536814008691/br2238Isup2.hkl

CCDC reference: 997763

Additional supporting information:  crystallographic information; 3D view; checkCIF report

## Figures and Tables

**Table 1:** Hydrogen-bond geometry (Å, °)

*D*—H⋯*A*	*D*—H	H⋯*A*	*D*⋯*A*	*D*—H⋯*A*
O4—H4⋯O7^i^	0.78 (8)	1.95 (8)	2.715 (5)	169 (7)
O8—H8⋯O3^ii^	0.87 (7)	1.8 (5)	2.712 (5)	175 (6)
O12—H12⋯O2	0.77 (8)	1.87 (8)	2.597 (5)	158 (10)
N1—H1*N*⋯O4^iii^	0.74 (6)	2.23 (6)	2.963 (6)	174 (7)
N1—H2*N*⋯O8^iv^	0.91 (8)	2.49 (7)	2.972 (6)	114 (6)
N1—H3*N*⋯O7	0.86 (6)	2.09 (7)	2.847 (5)	147 (8)
N1—H4*N*⋯O11^v^	0.95 (8)	2.11 (8)	3.056 (7)	172 (7)
N2—H1⋯O6^vi^	0.77 (7)	2.33 (8)	2.867 (3)	128 (9)
N2—H1⋯O8^vi^	0.77 (7)	2.59 (7)	3.236 (4)	143 (7)
N2—H2⋯O6^vii^	0.85 (9)	2.27 (10)	2.867 (3)	127 (8)
N2—H2⋯O10^vii^	0.85 (9)	2.55 (9)	3.371 (3)	163 (7)

Symmetry codes: (i) 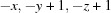; (ii) 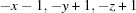; (iii) 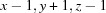; (iv) 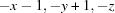; (v) 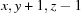; (vi) 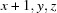; (vii) 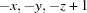.

**Fig. 1.**
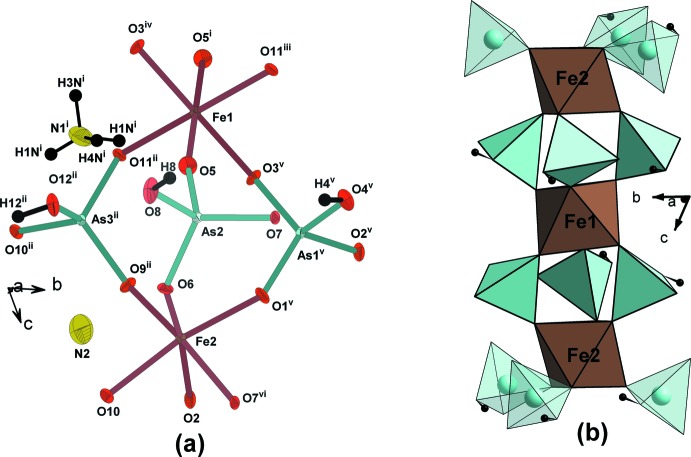
: (a) Unité asymétrique dans le composé α-NH4Fe(HAsO4)2. Les deux atomes d'hydrogène relatifs à N2, situé sur un centre d'inversion, ont été supprimés. Les éllipsoïdes ont été définis avec 50% de probabilité. [Symmetry codes: (i) -x, -y + 1, -z; (ii) -x, -y, -z + 1; (iii) x, y + 1, z - 1; (iv) x, y, z - 1; (v) -x, -y + 1, -z + 1; (vi) -x - 1, -y + 1, -z +1]. (b): Nouvelle unité centrosymétrique dans le composé α-NH4Fe(HAsO4)2.

**Fig. 2.**
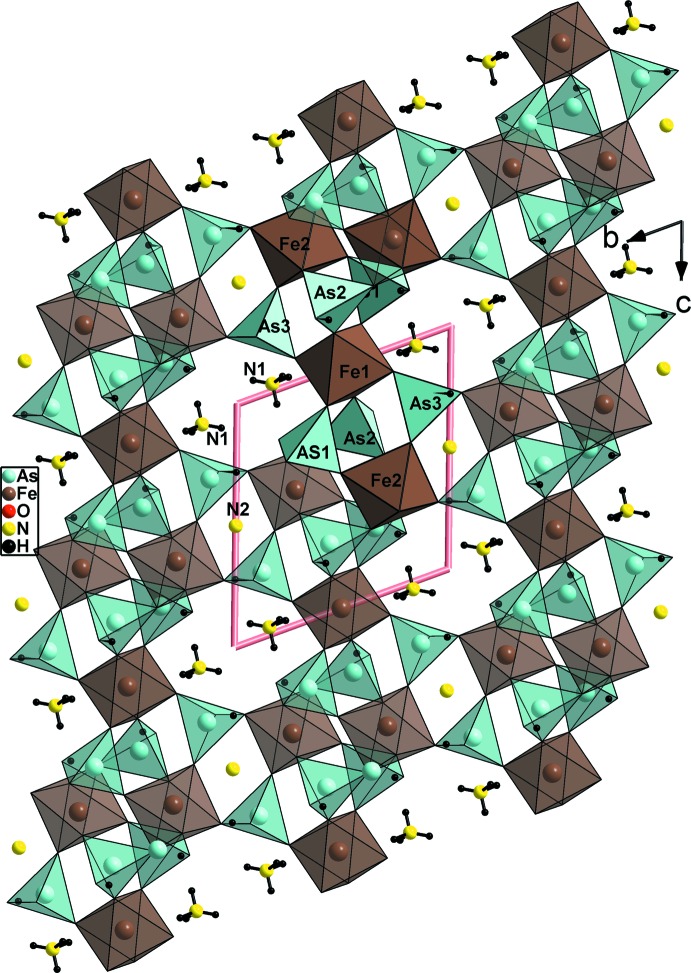
: Projection de la structure de α-NH4Fe(HAsO4)2 selon a, où l'unité centrosymétrique est indiquée en gras.

**Fig. 3.**
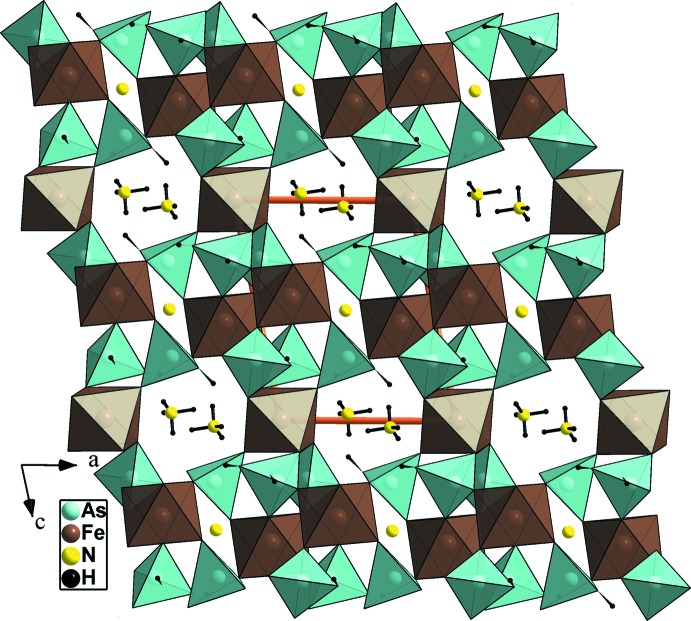
: Projection de la structure de α-NH4Fe(HAsO4)2 selon b.

**Fig. 4.**
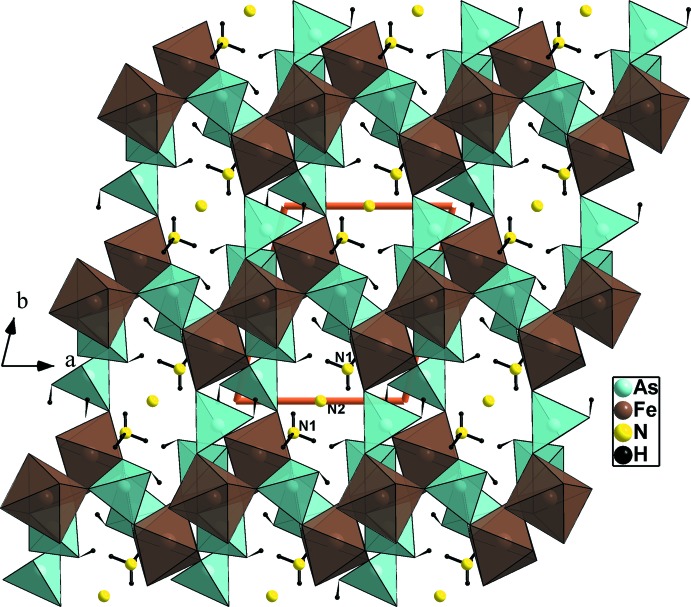
: Projection de la structure de α-NH4Fe(HAsO4)2 selon c.

**Fig. 5.**
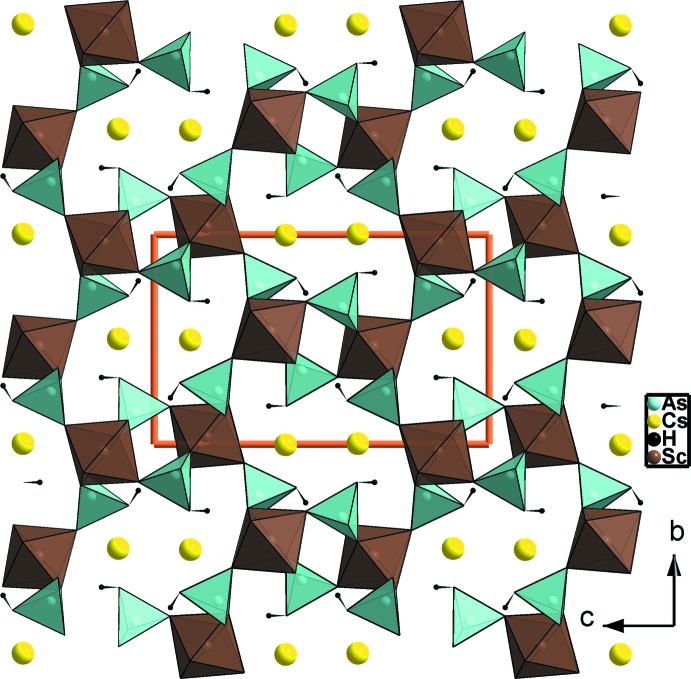
: Projection de la structure de β-CsSc(HAsO4)2 selon a.

**Fig. 6.**
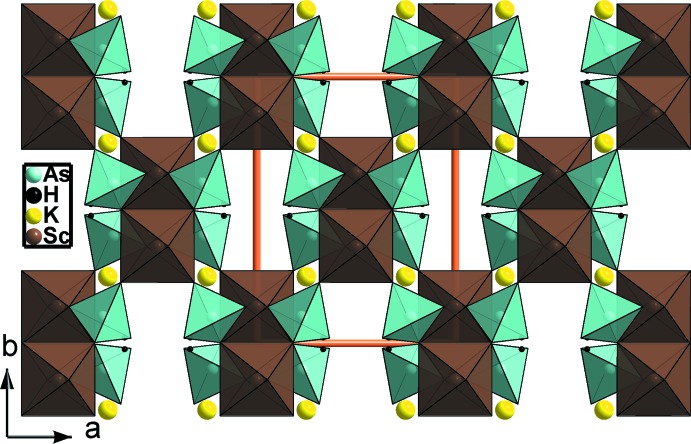
: Projection de la structure de KSc(HAsO4)2 selon c.

**Fig. 7.**
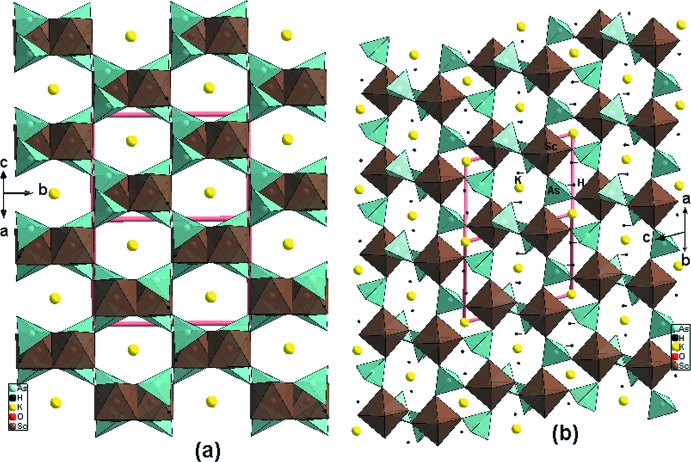
: (a): Projection de la structure de KSc(HAsO4)2 selon [-101], (b): Projection de la structure de KSc(HAsO4)2 selon [1-10].

## supplementary crystallographic information

### 1. Comment

*L*'un des principaux problèmes de la science et de la technologie moderne est la synthèse de nouveaux matériaux multifonctionnels. Ces dernières décennies, un grand intérêt est porté sur l'étude des phosphates acides de formulation générale *M*^I^*M*^III^(HPO_4_)_2_ qui sont caractérisés par leurs polymorphismes et leurs variétés structurelles. Vu l'importante ressemblance structurale entre les phosphates et les arséniates, et que ces derniers sont très peu étudiés (seuls trois composés ont été publiés). Nous avons choisis d'explorer le système arséniate acide de fer à cation monovalent. À cet égard, le présent travail porte sur l'étude structurale du premier arséniate acide de fer, α-NH_4_Fe(HAsO_4_)_2_.

Une étude bibliographique a été effectuée sur la famille de composés de formulation *M*^I^*M*^III^(HXO_4_)_2_ où A est un cation monovalent (Li, Na, K, Rb, Cs, Ag, NH_4_ et H_3_O); *M* est un m\'*et al.* trivalent (Fe, V, In, Ga, Al et Sc) et *X* peut être un phosphore ou un arsenic. D'après la littérature, les monophosphates de la famille *M*^I^*M*^III^(HPO_4_)_2_, cristallisent dans quatre systèmes cristallins à savoir, le triclinique, monoclinique, orthorhombique et le trigonal et adoptent six groupes d'espaces différents.

On dénombre essentiellement sept monophosphates cristallisant dans le système triclinique de groupe d'espace *P-1*, notés par la forme α. Ces composés sont NH_4_Fe(HPO_4_)_2_ (Yakubovich, 1993); α–RbFe(HPO_4_)_2_ et α–CsIn(HPO_4_)_2_ (Lesage *et al.*, 2007); α–RbV(HPO_4_)_2_ (Lii & Wu, 1994; Haushalter *et al.*, 1995; Lesage *et al.*, 2007); α–NH_4_V(HPO_4_)_2_ (Bircsak & Harrison, 1998); NH_4_(Al_0,64_Ga_0,36_)(HPO_4_)_2_ (Stalder & Wilkinson, 1998); (H_3_O)Al(HPO_4_)_2_ (Yan *et al.*, 2000).

Cristallisant dans le système monoclinique, groupe d'espace *P*2_1_/*c*, on peut citer les phosphates: β–RbV(HPO_4_)_2_ et β–NH_4_V(HPO_4_)_2_ (Haushalter *et al.*, 1995); β–CsIn(HPO_4_)_2_ (Huang *et al.*, 2004); CsSc(HPO_4_)_2_ (Menezes *et al.*, 2008); RbSc(HPO_4_)_2_ et (NH_4_)Sc(HPO_4_)_2_ (Menezes *et al.*, 2009*b*); (H_3_O)Fe(HPO_4_)_2_ (Vencato *et al.*, 1989); AIn(HPO_4_)_2_ (A= K, Rb, NH_4_ et Ag) (Mao *et al.*, 2002; Filaretov *et al.* 2002; Guesdon *et al.*, 2009) et finalement une phase non-centrosymétrique NaSc(HPO_4_)_2_ (Ewald *et al.*, 2004). Dans le système orthorhombique, on distingue deux phosphates: LiIn(HPO_4_)_2_ (*Pmnn*, Gurbanova *et al.*, 2001) et le KSc(HPO_4_)_2_ (*Pnma*; Menezes *et al.*, 2009*a*). Parmis cette famille de phosphates on rencontre trois composés cristallisant dans le système rhomboédrique notamment: RbFe(HPO_4_)_2_ (Lii & Wu, 1994; Villars *et al.*, 2007); RbGa(HPO_4_)_2_ et RbAl(HPO_4_)_2_ (Lesage *et al.*, 2007).

Comparés à leurs homologues phosphates, les monoarséniates sont très peu étudiés. Une étude bibliographique révèle seulement l'existence de trois composés. Ils cristallisent dans trois groupes d'espaces différents. Deux formes déjà vues dans les monophosphates, α-CsSc(HAsO4)_2_ et β-CsSc(HAsO4)_2_ (Schwendtner & Kolitsch, 2004*b*). La troisième forme représente une nouvelle structure non rencontrée dans les monphosphates, KSc(HAsO_4_)_2_ (Schwendtner & Kolitsch, 2004*a*). Elle cristallise dans le groupe d'espace *C*2/*c*.

La structure de α-NH_4_Fe(HAsO_4_)_2_, isotype à de nombreuses autres phases à savoir NH_4_Fe(HPO_4_)_2_ (Yakubovich, 1993) et α-CsSc(HAsO_4_)_2_ (Schwendtner & Kolitsch, 2004*b*). Elle est formée d'une charpente tridimensionnelle construite à partir des octaèdres FeO_6_ et des groupements hydroxyarséniates HAsO_4_ partageant des sommets oxygène.

L'unité asyétrique de la charpente anionique du composé étudié (Fig. 1a) est formée d'un cycle comprenant deux octaèdres FeO_6_ partageant chacun ses six sommets oxygène avec respectivement six tétraèdres HAsO_4_ différents. Le centre d'inversion situé sur l'atome Fe1 donne lieu à un dimère (nouvelle unité centrosymétrique de formule Fe_3_O_6_[HAsO_4_]_6_ (Fig. 1b).

Dans cette derrière l'octaèdre central Fe1O_6_ partage ses six sommets oxygène avec respectivement six tétraèdres HAsO_4_ de la même unité, alors que les deux autres octaèdres Fe2O_6_ partagent chacun trois de leurs sommets avec trois groupements hydroxyarséniates de la même unité. Les trois autres sommets sont mis en commun avec respectivement trois groupements appartenanant à trois unités adjacentes. Par cette disposition on remarque que chaque tétraèdre partage deux de ses sommets avec deux octaèdres de la même unité, le troisième avec un octaèdre d'une autre unité et le dernier sommet ne participe pas de manière directe à la charpente anionique mais il forme le groupement hydroxyle.

Ces unités centrosymétriques sont connectées selon la direction *c* (Fig. 2), formant ainsi des rubans [Fe_3_O_6_[HAsO_4_]_6_]_∞_. La jonction de ses rubans selon *a* et *b*, conduit à une charpente ouverte tridimensionnelle présentant de nombreux tunnels entrecroisés ou logent les cations ammonium (Fig. 2, 3 et 4).

Les figures 2 et 3 montrent l'existence de deux types de tunnels dirigés respectivement selon *a* et *b*. Dans le premier type, les tunnels présentent des sections internes délimitées par quatre octaèdres FeO_6_ et quatre tétraèdres de manière alternée, contenant les cations N1H_4_. Un deuxième type de tunnels, délimités par quatre arêtes appartenant à deux tétraèdres HAsO_4_ et deux octaèdres FeO_6_, contenant les cations N2H_4_^+^.

Un seul type de tunnel est observé selon la direction *c*. Ces derniers, contenant les cations N1H_4_ et N2H_4_ (Fig. 4), ont des sections plus larges que ceux observés selon les deux autres directions.

Les géométries des polyèdres sont conformes à celles rencontrées dans les structures de même formulation (Yakubovich, 1993; Lesage *et al.*, 2007; Schwendtner & Kolitsch 2004*a*, 2004*b*; Lii & Wu, 1994). Les valeurs moyennes pour les distances Fe–O sont 2,014 Å et 2,007 Å respectivement pour Fe1 et Fe2. Les distances moyennes As–O sont de 1,690 Å, 1,686 Å et 1,695 Å dans les groupements hydroxyarséniates respectivement As1, As2 et As3. La liaison la plus longue As–O, correspond au groupement hydroxyle. Ces derniers sont orientées selon les directions des atomes d'oxygène les plus proches pour former des liaisons hydrogène fortes. D'autre part, le calcul des valences de liaisons (BVS), utilisant la formule empirique de Brown (Brown & Altermatt, 1985), conduit aux valeurs des charges des cations suivants: Fe1(3,050), Fe2(3,089), As1(4,880), As2(4,937) et As3(4,821).

La géométrie des groupements ammonium est conforme avec celles rencontrées dans la littérature (Filaretov *et al.*, 2002; Yakubovich, 1993; Bircsak & Harrison, 1998; Stalder & Wilkinson, 1998; Haushalter *et al.*, 1995; Menezes *et al.*, 2009*b*).

Le degré global de distortion des différents polyèdres de coordination par rapport à leurs homologues réguliers est évalué à la fois par le nombre de coordination effectif ECoN (Nespolo, 2001 & Nespolo *et al.*, 2001) ainsi que les indices de distortion ID (Baur, 1974 & Wildner, 1992). Les groupements HAsO_4_ sont relativement réguliers ECoN ≈ 3,96. *L*'octaèdre Fe1O_6_ est légèrement distordu comparé à Fe2O_6_ avec ECoN (Fe2) 5,867 et ECoN (Fe1) 5,995. Les indices de distortion pour les différents polyèdres sont de l'ordre de 1 à 2%. Une légère distortion de 4% est relevée au niveau des angles O—Fe1—O et O—As1—O.

Le composé étudié α-NH_4_Fe(HAsO_4_)_2_ est isostructural à la forme α-CsSc(HAsO_4_)_2_ (Schwendtner & Kolitsch, 2004*b*). En effet, la comparaison de la structure avec celles de β-CsSc(HAsO_4_)_2_ et de KSc(HAsO_4_)_2_ (Schwendtner & Kolitsch, 2004*a*), montre que: dans le composé β-CsSc(HAsO_4_)_2_ (*P2_1_/c*), l'unité centrosymétrique est formée par quatre tétraèdres (HAsO_4_) et deux octaèdres ScO_6_. Ces unités se propagent selon [0-11] et [01-1] par mize en commun des sommets oxygène, formant ainsi une charpentre tridimentionnelle avec un seul type de tunnels larges parallèles à *a* où logent les cations monovalents (Fig. 5).

Dans le composé KSc(HAsO_4_)_2_ (*C2/c*), l'unité centrosymétrique est formée par deux tétraèdres et deux octaèdres de manière alternée (Fig. 6). *L*'enchainement de ces unités selon *c* par mize en commun des sommets oxygène, conduit à une charpente tridimensionnelle. Il apparait dans cette dernière, un premier type de tunnels selon [-101] de section relativement importante de forme hexagonale (Fig. 7a), deux autres de section moins importante sont dirigés selon [1-10] (Fig. 7b).

### 2. Experimental

La synthèse de la phase α-NH_4_Fe(HAsO_4_)_2_ a été réalisée par voie hydrothermale. Elle consiste à préparer une solution aqueuse contenant les réactifs suivants; 0,31 g de NaBO_3_·4H_2_O (FLUKA), 0,90 g de (NH_4_)_3_Fe(C_2_O_4_)_3_·3H_2_O (ACROS) et l'acide As_2_O_5_·7H_2_O (PROLABO) (quantité suffisante jusqu'à pH=1). Le mélange ainsi obtenu est légèrement chauffé sous agitation magnétique. On obtient alors une solution limpide de couleur verdâtre. Elle est placée ensuite dans un autoclave à 453 K. Des cristaux de forme parallélépipédique de couleur verdâtre apparaissent au bout de trois semaines.

### 3. Refinement

Les atomes autres que l'hydrogène ont éé affinés en anisotrope. Il en résulte des ellipsoïdes bien définis. Les positions des atomes H ont été déterminées par séries de Fourier-différence. Les distances O4—H4, O8—H8, O12—H12, N1—H2N, N1—H3N et N1—H4N, ont été fixées à 0,80 Å par l'option *DFIX* du programme *SHELXL97* (Sheldrick, 2008). N2 occupe un centre d'inversion, et par conséquent ses quatre protons doivent être en désordre sur au moins deux ensembles équivalents de sites. Les deux atomes d'hydrogène relatifs à N2, situé, sur un centre d'inversion, ont été par conséquent supprimés des figures. Les contraintes *U*_iso_(H) = 1,5 *U*_eq_(C) ont été appliquées pour les atomes H8, H2N, H3N et H4N. Les *U*_iso_ des atomes H restant ont été affinés sans contrainte. De plus, les densités d'électrons maximum et minimum restants dans la Fourier-différence sont acceptables et sont situées respectivements à 0,78 Å de O12 et à 0,91 Å de As2.

### Crystal data

**Table:**

(NH_4_)Fe(HAsO_4_)_2_	*Z* = 3
*M**_r_* = 353.75	*F*(000) = 507
Triclinic, *P*1	*D*_x_ = 3.236 Mg m^−^^3^
Hall symbol: -P 1	Mo *K*α radiation, λ = 0.71073 Å
*a* = 7.3473 (7) Å	Cell parameters from 25 reflections
*b* = 9.1917 (8) Å	θ = 11–15°
*c* = 9.7504 (9) Å	µ = 11.14 mm^−^^1^
α = 64.545 (5)°	*T* = 298 K
β = 70.710 (7)°	Prism, green
γ = 69.638 (6)°	0.21 × 0.13 × 0.09 mm
*V* = 544.55 (9) Å^3^	

### Data collection

**Table:**

Enraf–Nonius CAD-4 diffractometer	2121 reflections with *I* > 2σ(*I*)
Radiation source: fine-focus sealed tube	*R*_int_ = 0.021
Graphite monochromator	θ_max_ = 27.0°, θ_min_ = 2.4°
ω/2θ scans	*h* = −9→1
Absorption correction: ψ scan (North *et al.*, 1968)	*k* = −11→10
*T*_min_ = 0.187, *T*_max_ = 0.388	*l* = −12→11
2737 measured reflections	2 standard reflections every 120 min
2373 independent reflections	intensity decay: 1.1%

### Refinement

**Table:**

Refinement on *F*^2^	Secondary atom site location: difference Fourier map
Least-squares matrix: full	Hydrogen site location: inferred from neighbouring sites
*R*[*F*^2^ > 2σ(*F*^2^)] = 0.027	H atoms treated by a mixture of independent and constrained refinement
*wR*(*F*^2^) = 0.075	*w* = 1/[σ^2^(*F*_o_^2^) + (0.0471*P*)^2^ + 0.7636*P*] where *P* = (*F*_o_^2^ + 2*F*_c_^2^)/3
*S* = 1.05	(Δ/σ)_max_ < 0.001
2373 reflections	Δρ_max_ = 1.31 e Å^−^^3^
197 parameters	Δρ_min_ = −1.21 e Å^−^^3^
8 restraints	Extinction correction: *SHELXL97* (Sheldrick, 2008), Fc^*^=kFc[1+0.001xFc^2^λ^3^/sin(2θ)]^-1/4^
Primary atom site location: structure-invariant direct methods	Extinction coefficient: 0.0048 (7)

### Special details

**Table:**

Geometry. All e.s.d.'s (except the e.s.d. in the dihedral angle between two l.s. planes) are estimated using the full covariance matrix. The cell e.s.d.'s are taken into account individually in the estimation of e.s.d.'s in distances, angles and torsion angles; correlations between e.s.d.'s in cell parameters are only used when they are defined by crystal symmetry. An approximate (isotropic) treatment of cell e.s.d.'s is used for estimating e.s.d.'s involving l.s. planes.
Refinement. Refinement of *F*^2^ against ALL reflections. The weighted *R*-factor *wR* and goodness of fit *S* are based on *F*^2^, conventional *R*-factors *R* are based on *F*, with *F* set to zero for negative *F*^2^. The threshold expression of *F*^2^ > σ(*F*^2^) is used only for calculating *R*-factors(gt) *etc*. and is not relevant to the choice of reflections for refinement. *R*-factors based on *F*^2^ are statistically about twice as large as those based on *F*, and *R*- factors based on ALL data will be even larger.

### Fractional atomic coordinates and isotropic or equivalent isotropic displacement parameters (Å^2^)

**Table:**

	*x*	*y*	*z*	*U*_iso_*/*U*_eq_	
As1	0.09249 (5)	0.34877 (4)	0.71870 (4)	0.00566 (12)	
As2	−0.45289 (5)	0.44127 (5)	0.28294 (4)	0.00549 (12)	
As3	−0.07805 (5)	−0.12510 (5)	0.73233 (4)	0.00590 (12)	
Fe1	0.0000	0.5000	0.0000	0.00598 (17)	
Fe2	−0.20467 (8)	0.27790 (6)	0.55734 (6)	0.00606 (14)	
O1	0.1837 (4)	0.4902 (3)	0.5577 (3)	0.0124 (6)	
O2	−0.0136 (4)	0.2332 (3)	0.6883 (3)	0.0118 (6)	
O3	−0.0679 (4)	0.4228 (3)	0.8563 (3)	0.0106 (6)	
O4	0.2932 (4)	0.1977 (4)	0.7903 (4)	0.0144 (6)	
O5	−0.2458 (4)	0.4507 (4)	0.1459 (3)	0.0168 (6)	
O6	−0.4073 (4)	0.2983 (3)	0.4539 (3)	0.0097 (5)	
O7	−0.5723 (4)	0.6355 (3)	0.2809 (3)	0.0084 (5)	
O8	−0.5950 (5)	0.3629 (4)	0.2345 (4)	0.0183 (7)	
O9	−0.0227 (4)	−0.1830 (4)	0.5791 (3)	0.0121 (6)	
O10	−0.2573 (4)	0.0526 (3)	0.7080 (3)	0.0090 (5)	
O11	−0.1595 (4)	−0.2693 (3)	0.8989 (3)	0.0103 (6)	
O12	0.1255 (5)	−0.0844 (4)	0.7510 (4)	0.0149 (6)	
N1	−0.6133 (8)	0.8363 (6)	−0.0303 (6)	0.0304 (10)	
N2	0.5000	0.0000	0.5000	0.0386 (18)	
H4	0.371 (10)	0.243 (9)	0.782 (8)	0.05 (2)*	
H8	−0.707 (10)	0.427 (8)	0.210 (8)	0.058*	
H12	0.113 (14)	0.010 (8)	0.719 (10)	0.08 (3)*	
H1N	−0.644 (8)	0.926 (8)	−0.075 (7)	0.019 (15)*	
H2N	−0.644 (11)	0.751 (8)	−0.034 (8)	0.052*	
H4N	−0.474 (9)	0.793 (9)	−0.048 (8)	0.055*	
H3N	−0.626 (11)	0.814 (9)	0.067 (7)	0.055*	
H1	0.455 (12)	0.095 (7)	0.475 (9)	0.051*	
H2	0.437 (12)	−0.035 (11)	0.466 (9)	0.055*	

### Atomic displacement parameters (Å^2^)

**Table:**

	*U*^11^	*U*^22^	*U*^33^	*U*^12^	*U*^13^	*U*^23^
As1	0.0062 (2)	0.0047 (2)	0.0058 (2)	−0.00148 (14)	−0.00020 (14)	−0.00233 (15)
As2	0.0052 (2)	0.0054 (2)	0.0050 (2)	−0.00081 (14)	0.00030 (14)	−0.00243 (15)
As3	0.0065 (2)	0.00420 (19)	0.0058 (2)	−0.00022 (14)	−0.00039 (14)	−0.00223 (15)
Fe1	0.0067 (4)	0.0055 (4)	0.0040 (3)	−0.0008 (3)	0.0005 (3)	−0.0019 (3)
Fe2	0.0065 (3)	0.0055 (3)	0.0052 (3)	−0.0007 (2)	0.0000 (2)	−0.0025 (2)
O1	0.0149 (14)	0.0061 (13)	0.0104 (14)	−0.0042 (11)	0.0007 (11)	0.0011 (11)
O2	0.0157 (14)	0.0069 (13)	0.0166 (14)	−0.0025 (11)	−0.0078 (12)	−0.0045 (11)
O3	0.0096 (13)	0.0125 (14)	0.0108 (14)	0.0011 (11)	−0.0007 (11)	−0.0092 (12)
O4	0.0094 (14)	0.0092 (14)	0.0209 (16)	0.0003 (11)	−0.0065 (12)	−0.0018 (12)
O5	0.0137 (15)	0.0158 (15)	0.0149 (15)	−0.0045 (12)	0.0064 (12)	−0.0062 (13)
O6	0.0122 (13)	0.0104 (13)	0.0060 (12)	−0.0028 (11)	−0.0046 (10)	−0.0005 (10)
O7	0.0094 (13)	0.0048 (12)	0.0079 (13)	−0.0007 (10)	0.0022 (10)	−0.0033 (11)
O8	0.0156 (15)	0.0141 (15)	0.0337 (19)	0.0005 (12)	−0.0142 (14)	−0.0126 (14)
O9	0.0125 (14)	0.0144 (14)	0.0104 (13)	−0.0013 (11)	0.0004 (11)	−0.0089 (12)
O10	0.0064 (12)	0.0064 (12)	0.0093 (13)	0.0012 (10)	−0.0011 (10)	−0.0011 (11)
O11	0.0125 (13)	0.0062 (13)	0.0066 (13)	−0.0009 (11)	0.0009 (10)	−0.0003 (11)
O12	0.0144 (14)	0.0074 (14)	0.0250 (17)	−0.0016 (12)	−0.0086 (13)	−0.0052 (13)
N1	0.038 (3)	0.021 (2)	0.018 (2)	0.003 (2)	−0.008 (2)	−0.0013 (19)
N2	0.054 (5)	0.030 (4)	0.040 (4)	−0.028 (4)	−0.003 (4)	−0.011 (4)

### Geometric parameters (Å, º)

**Table:**

As1—O1	1.661 (3)	Fe2—O1^v^	1.971 (3)
As1—O2	1.680 (3)	Fe2—O6	1.976 (3)
As1—O3	1.683 (3)	Fe2—O2	2.025 (3)
As1—O4	1.735 (3)	Fe2—O10	2.040 (3)
As2—O5	1.660 (3)	Fe2—O7^vi^	2.067 (3)
As2—O6	1.673 (3)	O1—Fe2^v^	1.971 (3)
As2—O7	1.688 (3)	O3—Fe1^vii^	2.068 (3)
As2—O8	1.724 (3)	O4—H4	0.78 (6)
As3—O9	1.677 (3)	O7—Fe2^vi^	2.067 (3)
As3—O11	1.677 (3)	O8—H8	0.87 (6)
As3—O10	1.683 (3)	O9—Fe2^ii^	1.959 (3)
As3—O12	1.743 (3)	O11—Fe1^viii^	2.020 (3)
Fe1—O5^i^	1.952 (3)	O12—H12	0.77 (7)
Fe1—O5	1.952 (3)	N1—H1N	0.74 (6)
Fe1—O11^ii^	2.020 (3)	N1—H2N	0.90 (6)
Fe1—O11^iii^	2.020 (3)	N1—H4N	0.95 (6)
Fe1—O3^iv^	2.068 (3)	N1—H3N	0.86 (6)
Fe1—O3^v^	2.068 (3)	N2—H1	0.77 (6)
Fe2—O9^ii^	1.959 (3)	N2—H2	0.85 (6)
			
O1—As1—O2	114.30 (15)	O9^ii^—Fe2—O6	95.61 (12)
O1—As1—O3	115.11 (14)	O1^v^—Fe2—O6	94.53 (12)
O2—As1—O3	108.14 (14)	O9^ii^—Fe2—O2	87.36 (12)
O1—As1—O4	107.20 (14)	O1^v^—Fe2—O2	92.29 (12)
O2—As1—O4	100.18 (14)	O6—Fe2—O2	172.23 (12)
O3—As1—O4	110.91 (14)	O9^ii^—Fe2—O10	93.83 (11)
O5—As2—O6	111.33 (15)	O1^v^—Fe2—O10	169.74 (12)
O5—As2—O7	108.39 (14)	O6—Fe2—O10	87.51 (11)
O6—As2—O7	116.50 (13)	O2—Fe2—O10	85.13 (11)
O5—As2—O8	106.49 (16)	O9^ii^—Fe2—O7^vi^	174.07 (12)
O6—As2—O8	103.50 (15)	O1^v^—Fe2—O7^vi^	86.46 (11)
O7—As2—O8	110.12 (14)	O6—Fe2—O7^vi^	89.57 (11)
O9—As3—O11	111.89 (14)	O2—Fe2—O7^vi^	87.14 (12)
O9—As3—O10	109.73 (14)	O10—Fe2—O7^vi^	83.49 (11)
O11—As3—O10	108.13 (13)	As1—O1—Fe2^v^	149.07 (18)
O9—As3—O12	111.73 (15)	As1—O2—Fe2	135.82 (16)
O11—As3—O12	108.73 (14)	As1—O3—Fe1^vii^	127.03 (15)
O10—As3—O12	106.41 (14)	As1—O4—H4	107 (6)
O5^i^—Fe1—O5	180.00 (19)	As2—O5—Fe1	166.94 (19)
O5^i^—Fe1—O11^ii^	88.09 (12)	As2—O6—Fe2	126.17 (16)
O5—Fe1—O11^ii^	91.91 (12)	As2—O7—Fe2^vi^	131.78 (15)
O5^i^—Fe1—O11^iii^	91.91 (12)	As2—O8—H8	118 (5)
O5—Fe1—O11^iii^	88.09 (12)	As3—O9—Fe2^ii^	141.22 (17)
O11^ii^—Fe1—O11^iii^	180.00 (17)	As3—O10—Fe2	122.77 (14)
O5^i^—Fe1—O3^iv^	92.76 (12)	As3—O11—Fe1^viii^	126.26 (15)
O5—Fe1—O3^iv^	87.24 (12)	As3—O12—H12	109 (7)
O11^ii^—Fe1—O3^iv^	91.51 (11)	H1N—N1—H2N	128 (6)
O11^iii^—Fe1—O3^iv^	88.49 (11)	H1N—N1—H4N	114 (6)
O5^i^—Fe1—O3^v^	87.24 (12)	H2N—N1—H4N	97 (6)
O5—Fe1—O3^v^	92.76 (12)	H1N—N1—H3N	114 (7)
O11^ii^—Fe1—O3^v^	88.49 (11)	H2N—N1—H3N	105 (7)
O11^iii^—Fe1—O3^v^	91.51 (11)	H4N—N1—H3N	94 (6)
O3^iv^—Fe1—O3^v^	180.00 (11)	H1—N2—H2	104 (6)
O9^ii^—Fe2—O1^v^	95.98 (12)		

Symmetry codes: (i) −*x*, −*y*+1, −*z*; (ii) −*x*, −*y*, −*z*+1; (iii) *x*, *y*+1, *z*−1; (iv) *x*, *y*, *z*−1; (v) −*x*, −*y*+1, −*z*+1; (vi) −*x*−1, −*y*+1, −*z*+1; (vii) *x*, *y*, *z*+1; (viii) *x*, *y*−1, *z*+1.

### Hydrogen-bond geometry (Å, º)

**Table:**

*D*—H···*A*	*D*—H	H···*A*	*D*···*A*	*D*—H···*A*
O4—H4···O7^v^	0.78 (8)	1.95 (8)	2.715 (5)	169 (7)
O8—H8···O3^vi^	0.87 (7)	1.8 (5)	2.712 (5)	175 (6)
O12—H12···O2	0.77 (8)	1.87 (8)	2.597 (5)	158 (10)
N1—H1*N*···O4^ix^	0.74 (6)	2.23 (6)	2.963 (6)	174 (7)
N1—H2*N*···O8^x^	0.91 (8)	2.49 (7)	2.972 (6)	114 (6)
N1—H3*N*···O7	0.86 (6)	2.09 (7)	2.847 (5)	147 (8)
N1—H4*N*···O11^iii^	0.95 (8)	2.11 (8)	3.056 (7)	172 (7)
N2—H1···O6^xi^	0.77 (7)	2.33 (8)	2.867 (3)	128 (9)
N2—H1···O8^xi^	0.77 (7)	2.59 (7)	3.236 (4)	143 (7)
N2—H2···O6^ii^	0.85 (9)	2.27 (10)	2.867 (3)	127 (8)
N2—H2···O10^ii^	0.85 (9)	2.55 (9)	3.371 (3)	163 (7)

Symmetry codes: (ii) −*x*, −*y*, −*z*+1; (iii) *x*, *y*+1, *z*−1; (v) −*x*, −*y*+1, −*z*+1; (vi) −*x*−1, −*y*+1, −*z*+1; (ix) *x*−1, *y*+1, *z*−1; (x) −*x*−1, −*y*+1, −*z*; (xi) *x*+1, *y*, *z*.

### Distortion parameters ECoN and ID for the coordination polyhedra around Fe and As.

**Table:**

Polyedra	ECoN	ID_d_	ID_a_	ID_o_
Fe1O_6_	5.869	0.019	0.042	0.023
Fe2O_6_	5.910	0.020	0.023	0.018
As1O_4_	3.963	0.013	0.038	0.021
As2O_4_	3.969	0.012	0.023	0.019
As3O_4_	3.964	0.014	0.016	0.010

ID_d_=[Σ_i=1_^n1^(|d_i_-d_m_|)/n_1_d_m_]; ID_a_=[Σ_i=1_^n2^(|a_i_-a_m_|)/n_2_a_m_] and ID_o_=[Σ_i=1_^n2^(|o_i_-o_m_|)/n_2_o_m_].d,a, o signify Fe/As-O bond distance, O-Fe/As-O angle and O-O edge within the relevant polyhedron; index i indicates individual values, index m the mean value for the polyhedron. n1 and n2 are 4 and 6 for the arsenate tetrahedra and 6 and 12 for the iron octahedral.
